# Operative R0 resection of diffuse large B-cell lymphoma of the pelvis: a case report

**DOI:** 10.1186/s13256-018-1838-1

**Published:** 2018-10-13

**Authors:** Valerie Wienerroither, Martin Sauerschnig, Christine Beham-Schmid, Erwin Mathew, Azab El-Shabrawi, Hans-Jörg Mischinger, Peter Kornprat

**Affiliations:** 1 Department of General Surgery, Medical University of Graz, Auenbruggerplatz 29, 8036 Graz, Austria; 2 Ludwig Boltzmann Institute for Experimental and Clinical Traumatology in the AUVA Trauma Research Center, Austrian Cluster for Tissue Regeneration, Donaueschingerstraße 13, Vienna, 1200 Austria; 3 Institute of Pathology, Medical University of Graz, Neue Stiftingtalstraße 6, Graz, 8010 Austria

**Keywords:** Expansion of the pelvis, Diffuse large B-cell lymphoma, Intestinal perforation, R-CHOP

## Abstract

**Background:**

Diffuse large B-cell lymphoma is the most common subtype of non-Hodgkin lymphoma with or without involvement of extranodal sites. Rituximab in combination with cyclophosphamide, doxorubicin, vincristine and prednisolone (R-CHOP) therapy represents the current standard therapy, achieving a rather dissatisfying outcome in approximately 30–40% of all cases.

**Case presentation:**

We present the case of a 43-year-old Austrian woman with an incidentally detected large pelvic mass which was diagnosed as diffuse large B-cell lymphoma. Initially, the lymphoma intraoperatively appeared to be an inoperable conglomerate tumor. Soon, intestinal perforation induced by tumor infiltration occurred, which initiated a closure of the small intestine and application of a jejunal probe and a percutaneous endoscopic gastrotomy tube. Treatment utilizing the gold standard rituximab in combination with cyclophosphamide, doxorubicin, vincristine and prednisolone (R-CHOP) was performed, partly resulting in remission according to radiological follow-up. In view of diagnosis and primary treatment development, the predictive outcome appeared unsound. However, within the procedure of the latest surgical intervention, which was intended to at least reconstruct the intestinal passage in order to improve quality of life, a surgical R0 resection of the residual tumor mass was achieved.

**Conclusions:**

The case presented here reports an unanticipated process of diffuse large B-cell lymphoma, underlining the importance of interdisciplinary cooperation and surgical intervention within the realms of state-of-the-art treatment.

## Background

Diffuse large B-cell lymphoma (DLBCL) represents the most common subtype of non-Hodgkin lymphoma (NHL) with a proportion of approximately 30% of this entity [1] and occurs as a high-grade lymphoma with an incidence of 3.8/100,000 per year in Europe [2]. In the United States of America (USA), NHL in general accounts for 3.4% of cancer-related deaths [3–5] and for 3.06% in Europe [6].

Concerning DLBCL, the median age of patients ranges between the sixth and seventh decade [7]. Involvement of extranodal sites appears in 30% of all cases [8]; the gastrointestinal tract represents the most frequent localization.

Although the specific cause of DLBCL remains unclear, there are known risk factors including family history of hematologic malignancy, autoimmune disease, hepatitis C virus (HCV) seropositivity, atopic disease, and adiposity in youth [9].

R-CHOP (that is, rituximab, cyclophosphamide, doxorubicin, vincristine, and prednisone) is the most frequently applied treatment in DLBCL and provides the global gold standard while alternative strategies are sparse.

Despite variable therapeutic success, 30–40% of patients treated with R-CHOP cannot be cured and consequently suffer relapse [10] and death [5].

The 5-year progression-free survival (PFS) is between 80 and 85% in patients at a limited stage of disease, while patients with advanced disease show a dramatic decrease of PFS down to 50% [7].

The present case report describes the rare case of a woman with an initially inoperable form of DLBCL with primary subtotal affection of her urogenital system, who underwent standard R-CHOP. The therapeutic regimen delivered marginal tumor mass remission, but the case could then be unexpectedly resolved in a surgical procedure achieving R0 resection.

## Case presentation

After the incidental sonographic finding of a huge inhomogeneous expansion near the ovarian fossa, a 43-year-old, previously healthy, Austrian woman was referred to our university hospital. She reported a slight performance weakness, but denied any abdominal pain. Her medical history was unremarkable. She was a mother of two children, both by spontaneous vaginal delivery. Her abdominal surgical history included only an open appendectomy in childhood. She had no significant family or psychosocial history. She did not take any medication and denied tobacco smoking and alcohol intake. She was married and worked as a child carer. A clinical examination at the initial presentation showed a well-oriented, apyretic patient. Her vital signs were stable with a blood pressure of 135/70, a pulse rate of 73 beats/minute, and a body temperature of 36.4 °C. In abdominal examination, a poorly displaceable mass in her lower abdomen was palpable. Further physical examination showed no other abnormalities. A neurological examination was unremarkable. Laboratory findings (including complete blood count, liver function, renal function, coagulation status, and C-reactive protein) were within normal ranges, except for a slightly raised level of creatinine (1.02 mg/dL).

**Fig. 1:**
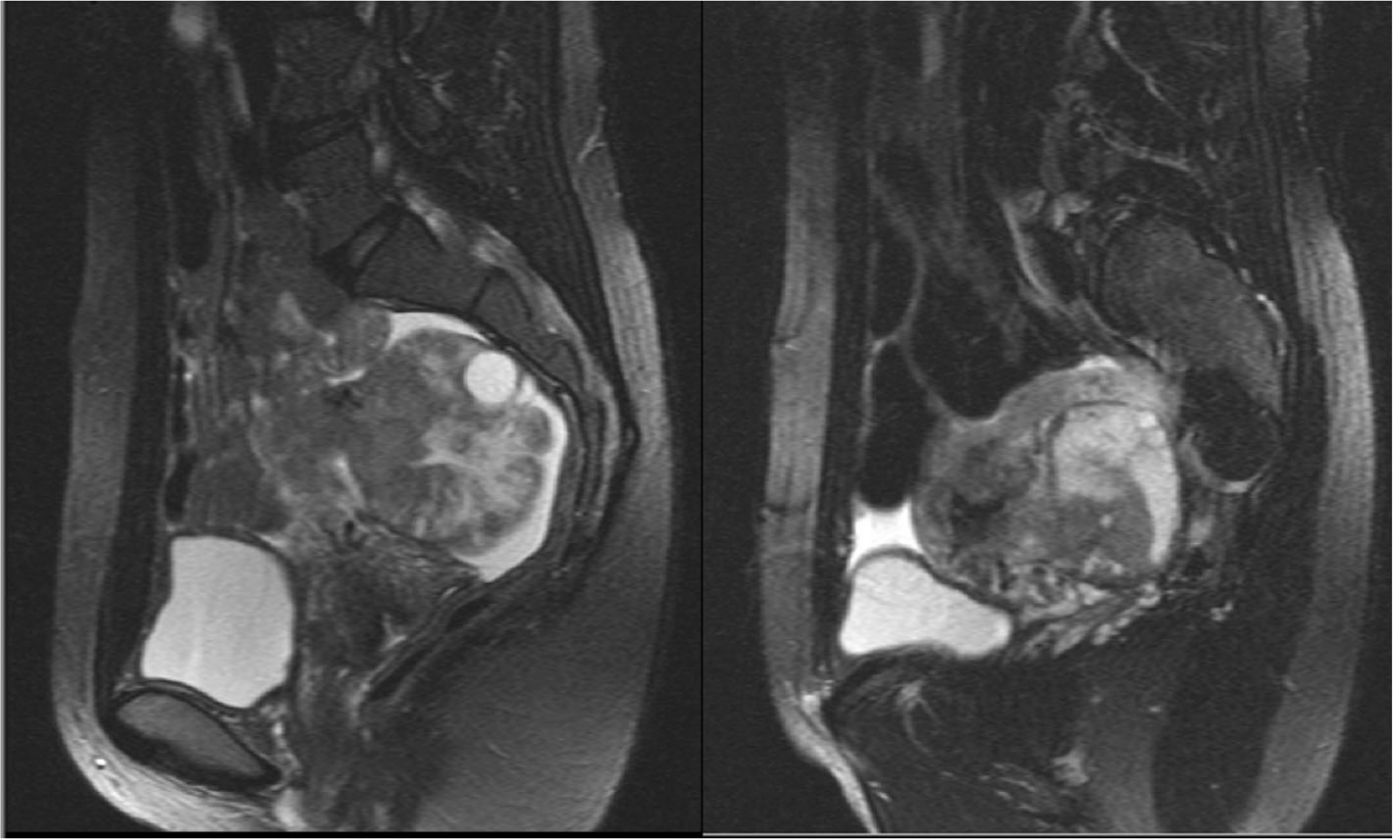
Magnetic resonance imaging of the pelvis. Magnetic resonance imaging showed an inhomogeneous tumor mass in the lesser pelvis with a size of 11.4 × 8.6 × 11.7 cm

A performed magnetic resonance imaging (MRI) showed a partially solid tumor within the lesser pelvic region with a size of 11.4 × 8.6 × 11.7 cm that infiltrated her left ureter, which resulted in consecutive urine retention and eventually in third grade hydronephrosis of her left kidney (Fig. 1).

**Fig. 2:**
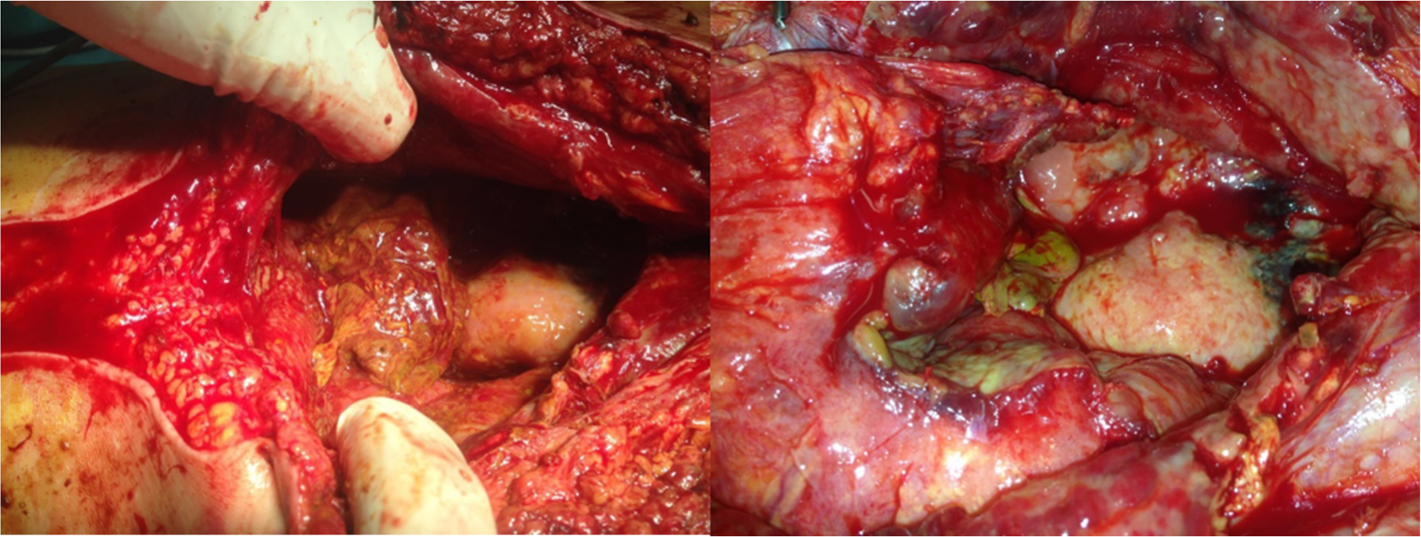
Tumor; intraoperative examination. Intraoperative – lesser pelvis with view of the tumor mass

Then, an explorative laparotomy was applied, in which a conglomerate tumor of unknown primary origin with deteriorating affection of uterus and left adnexa was confirmed, which was adherent to the pelvic wall and musculature of her retroperitoneum (Fig. 2). The radiologically detected infiltration of her left ureter and her urinary bladder was also verified intraoperatively. A rapid histopathological incision examination of intraoperatively sampled tumor tissue was prepared, which yielded an uncertain status with assumed leiomyosarcoma. For treatment of the left-sided urinary stasis, a ureteral stent was placed, which postoperatively had to be relocated by nephrostomy. According to interdisciplinary consultation following the stated initial surgery, the tumor had to be classified as inoperable.

**Fig. 3:**
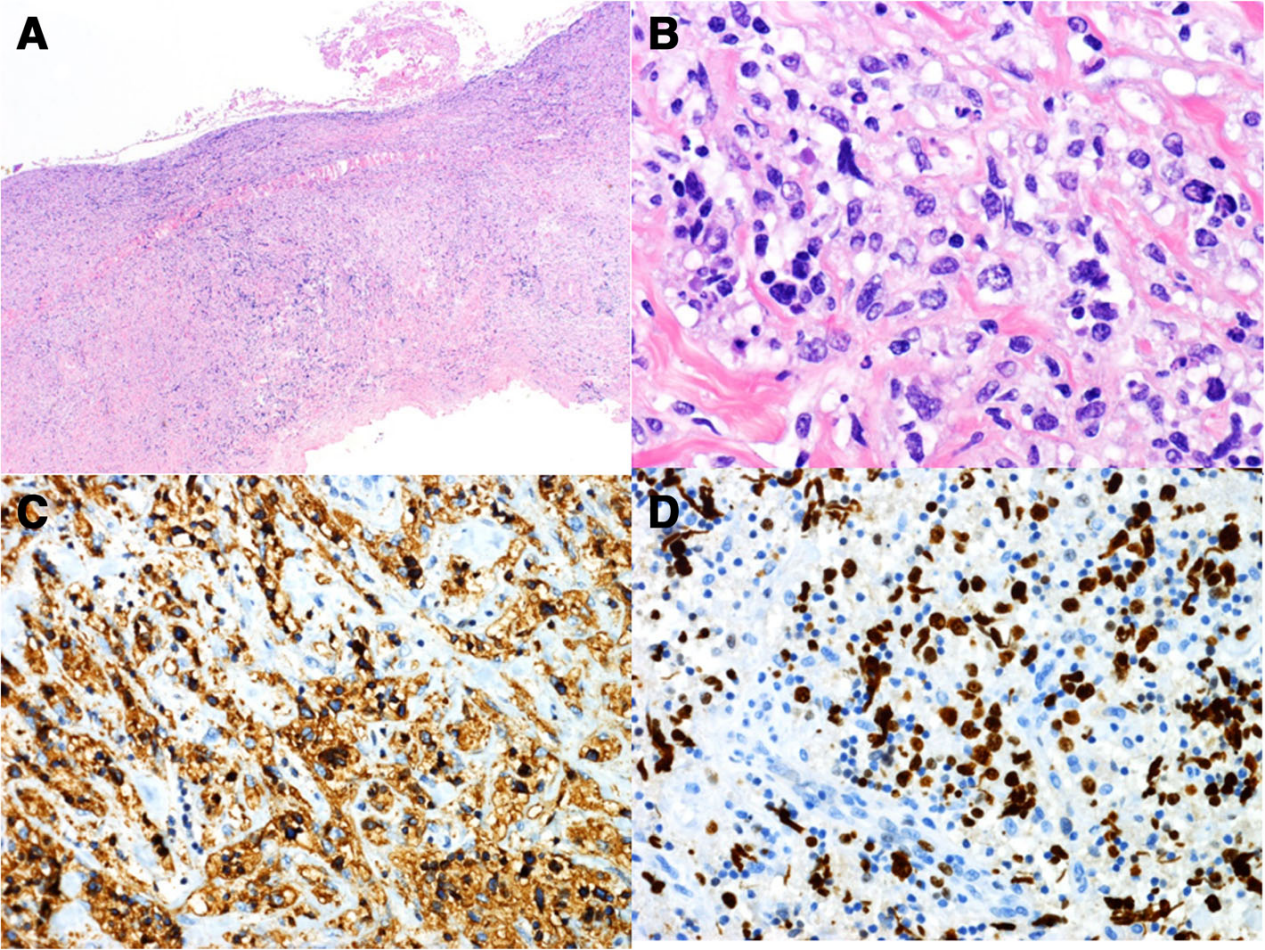
Resected tissue; microscopic examination. Histological work-up shows hyalinized and sclerosed peritoneal tissue diffusely infiltrated by polymorphous blasts with one or more nuclei (**a**, **b**; hematoxylin and eosin). Immunohistochemical staining showed a strong CD20 positivity of the blasts (**c**). The proliferation fraction using an antibody to Ki67 was 70% within the blast cell population (**d**)

Extended immunohistological analysis revealed DLBCL of the activated B cell-like (ABC) subtype with an International Prognostic Index (IPI) 1 and tumor cells positive for CD20 and CD79a (Fig. 3).

A biopsy was conducted followed by histological and cytological investigation; they did not detect any involving affection or infiltration of the bone marrow.

Fluorodeoxyglucose-positron emission tomography (FDG-PET) showed bone marrow activation among the central region without significant focal accentuation.

In an interdisciplinary tumor board, a therapeutic regime for our patient with a potential curative therapy objective utilizing R-CHOP was determined.

After beginning the treatment, an intestinal perforation occurred that induced another surgical intervention. On intraoperative examination, peritonitis as well as massive intraabdominal adhesions in terms of a *frozen abdomen* could be verified. The small intestine itself was found to be partly necrotic and obviously infiltrated by the lymphoma. The necrotic intestinal parts were resected, but due to massive peritonitis neither creation of ostomy nor enteroanastomosis was feasible. Therefore, a closure of the small intestine was performed and a jejunal probe as well as a percutaneous endoscopic gastrostomy (PEG) tube were installed.

Among further exploration, a colon fistula was identified and sewed over. Blood cultures were taken at varying times and were prepared according to the standard procedure. All of these cultures yielded negative results.

Chemotherapy was then proceeded with the objective to reduce tumor mass and enable a following reconstruction of the intestinal passage.

However, after four cycles of R-CHOP, only a slight tendency toward remission could be achieved, which provided a quite limited treatment effect. Thus, a histological examination of the residual tumor by surgical intervention was recommended by hematologist-oncologists. As her poor general condition did not allow higher doses of chemotherapy, a continuation of the current therapy with R-CHOP was determined, despite only partial remission. After a total of eight cycles of R-CHOP, re-surgery addressing enteroanastomosis to enable self-administered food intake and to improve quality of life as far as possible was performed. Furthermore, according to consensus of the institutional tumor board, another biopsy of the residual tumor and further histopathological analyses were conducted.

At the time of the stated intervention, she had been parenterally fed for 8 months already.

After this long period, the creation of a re-anastomosis seemed doubtful.

Intraoperative findings confirmed a conglomerate tumor imposing deteriorating effects on her jejunum, her sigmoid, her left adnexa, and part of her uterus. Extended inspection of the morphological aspects of the tumor mass on sight and pondering surgical/technical possibilities resulted in the rather unforeseen decision to attempt a resection of the tumor mass as a whole, accompanied by the reconstructive procedures outlined above.

**Fig. 4:**
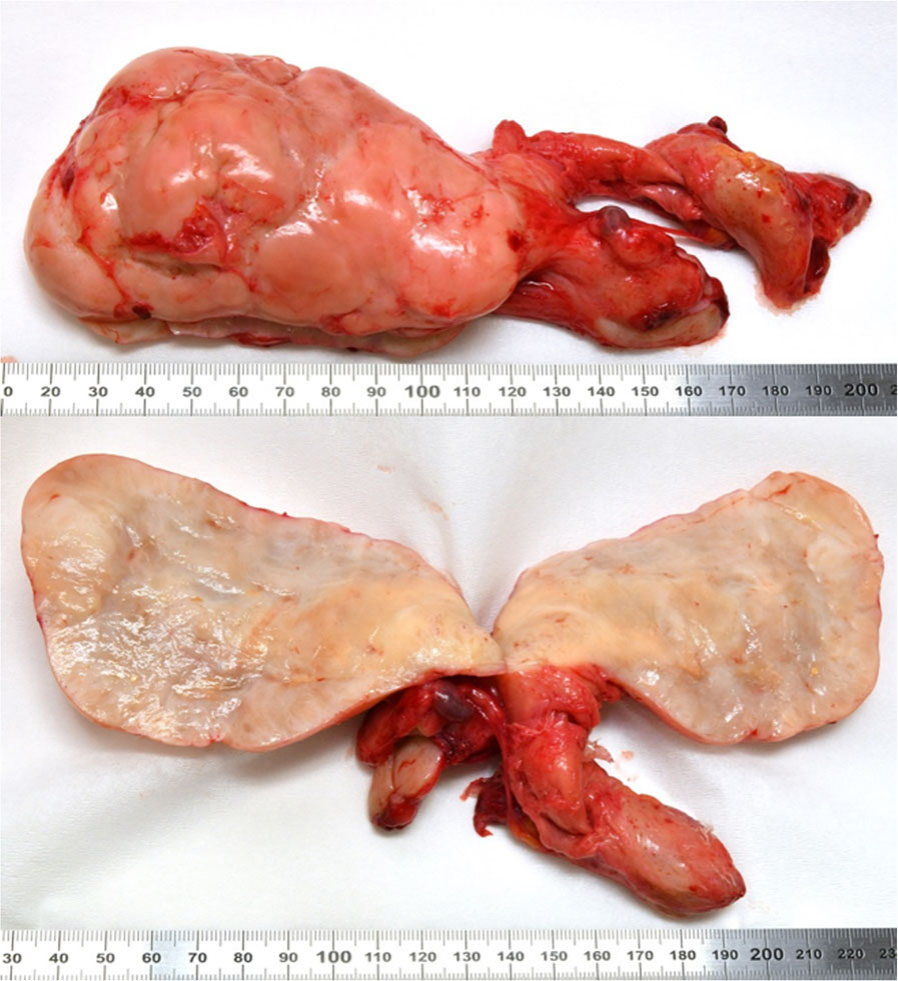
Resected tissue; macroscopic examination. Macroscopic view of the resected remnant tumor

**Fig. 5:**
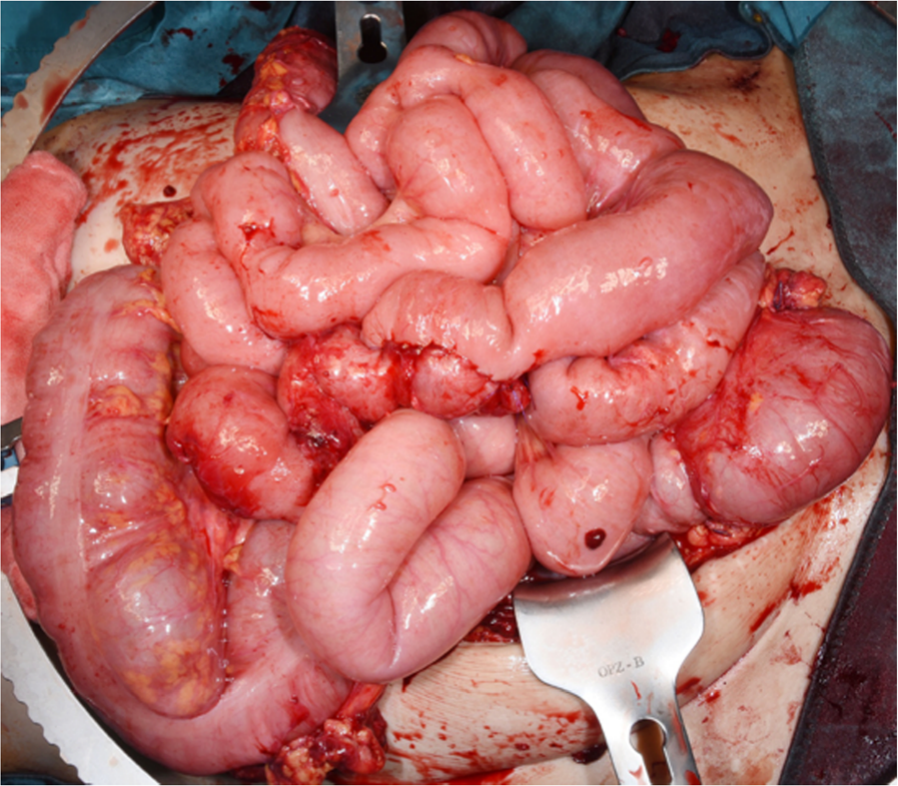
Intestine and anastomosis. Intraoperative view of the intestine after anastomosis

Thus, a complete resection of the tumor according to this intraoperative decision by entrainment of the left adnexa and part of her uterus could finally be implemented. A rapid histopathological incision examination confirmed R0 resection (Fig. 4). Against the odds, the residual smaller bowel appeared recovered and therefore both a successful end-to-end jejunostomy as well as a descendosigmoidostomy were implied (Fig. 5).

The recovery period passed uneventfully and shortly after, she could be released from hospital in good condition to out-patient follow-up treatment.

A full remission could be determined and close-meshed follow-up inspections were carried out.

In these follow-up inspections, no evidence of relapse was found so that at least 7 months of PFS and 7 months overall survival (OS) after R0 resection could be registered. An abdominal sonography without pathological findings was conducted 6 months after the last surgery.

## Discussion

The reported case describes an uncommon progression of extranodal DLBCL with an unusual reaction to standard treatment. Despite bad prognosis, unexpected remission could be reached via a delicate decision that was made spontaneously during a surgical intervention. Prior to the latter surgery, our patient’s digestive tract had to be bypassed for 8 months, due to intermediately occurring complications. Against expectations, intestinal passage could be successfully enabled again after such a long period of parenteral feeding. There are severe cases of DLBCL of the pelvis reported in the literature, but to the best of our knowledge this is the first case of DLBCL that evinces this exceptional complexity, an extraordinary course of disease, and describes an unconventional treatment that led to full remission.

DLBCL represents the most common type of NHL and constitutes approximately 30% of cases [1]. Extranodal appearance occurs in approximately 30% of these instances [8]. R-CHOP, which was chosen as first-line treatment in our case, is still the acknowledged standard of care in DLBCL [5, 11, 12]. As in the case reported here, younger patients in particular show significantly better outcomes when rituximab is combined with CHOP, as compared to controls treated with CHOP alone [13]. However, only a slight majority of patients burdened by DLBCL can be successfully treated by R-CHOP therapy and benefits differ in dependency of individual patients and even because of unknown factors [14].

For stratifying treatment strategies, a scoring according to the revised IPI (R-IPI) is seen as useful [15, 16]. R-IPI includes age, Eastern Cooperative Oncology Group performance status, serum lactate dehydrogenase (LDH) level, number of extranodal sites, and stage as variables [10]. According to these parameters, patients with DLBCL can be stratified in four risk groups. The patient in our case report was classified as R-IPI 1, meaning low risk. However, the therapy process turned out to be partially insufficient. This may be caused by diverse molecular subtypes [17], which was determined as ABC in this case.

The molecular heterogeneity of DLBCL is generally known to cause differences in outcome after R-CHOP therapy [18]. DLBCL can be distinguished in molecular subtypes by applying the cell of origin classification, in which the tumor profiles are compared to profiles of normal B cells [19]. Germinal center B cell-like (GBC) DLBCL express genes found in normal germinal center B cells and can be distinguished from ABC DLBCL, which are derived from antigen-activated B cells [20]. Approximately 15% are declared as unclassifiable DLBCL [21]. This classification shows significant differences in OS after treatment with R-CHOP, where therapy seems less effective in ABC DLBCL [21].

The case presented here points out an example of extranodal ABC DLBCL affecting the realms of the urogenital region burdened with an extraordinary condition, which was finally redeemed via re-assessed surgical intervention. State-of-the-art chemotherapy conducted at the beginning only delivered a merely discrete remission of the tumor mass. On grounds of the facts given at this point, surgical intervention was planned on the basis of palliative considerations.

Despite unsatisfying outcome of R-CHOP according to radiological follow-up, a surgical intervention would, however, hold the promise of a favorable deviation from the prospective outcome. This example shows the importance of reevaluation of surgical treatment possibilities, namely on-sight within the intraoperative setup through very critical reevaluation and inspection. Consecutive decision making might then even imply unexpected aspects that may lead toward the option of R0 resection; confirmed via rapid excision examination. All of this may not fully correspond to interdisciplinary guidelines that are often hampered by the growing influence of external factors, occasionally even of an economic nature (that is, sophisticated procedures, surgery time, and so on). Regarding the aspects of lymphoma and related pathologies, the latter mentioned of those contingencies can unfortunately never be predicted with certainty. This limitation may, on the other hand, sometimes be associated with unusual conditions and therefore unusual treatment options that should be considered. The reported unusual course of disease with an uncommon reaction to standard treatment in combination with extraordinary healing after many complications, and the surprising cure after anomalous spontaneous intraoperative decision makes this case unique and it differs from what we already know about DLBCL.

## Conclusions

As R-CHOP, which is the standard treatment of DLBCL, often results in unfavorable outcomes, tumor boards may decide to refrain from further surgical intervention or limit themselves to palliative procedures only. Detailed and unprejudiced reevaluation of every single feasible option – even including highly sophisticated and potentially non-economic, time-consuming surgical possibilities – should be strongly considered in order to achieve outstanding results of treatment and care.

## Abbreviations

ABC: Activated B cell-like
DLBCL: Diffuse large B-cell lymphoma
FDG-PET: Fluorodeoxyglucose-positron emission tomography
GBC: Germinal center B cell-like
IPI: International Prognostic Index
LDH: Lactate dehydrogenase
MRI: Magnetic resonance imaging
NHL: Non-Hodgkin lymphoma
OS: Overall survival
PEG: Percutaneous endoscopic gastrostomy
PFS: Progression-free survival
R-CHOP: Rituximab, cyclophosphamide, doxorubicin, vincristine, and prednisone
R-IPI: Revised International Prognostic Index
